# Mechanisms of metabolic reprogramming in abdominal aortic aneurysm

**DOI:** 10.3389/fcell.2025.1718220

**Published:** 2025-11-13

**Authors:** Xiaodong Wu, Jianpeng Li, Qiaoyue Ju, Yuqi Zeng, Junjie Cao

**Affiliations:** 1 Department of Vascular Surgery, Taizhou Second People’s Hospital Affiliated to Yangzhou University, Taizhou, Jiangsu, China; 2 Department of Cardiology, Taizhou Second People’s Hospital Affiliated to Yangzhou University, Taizhou, Jiangsu, China; 3 Operating room, Suzhou BenQ Medical Center, Suzhou, Jiangsu, China; 4 Department of Vascular Surgery, The Affiliated Suzhou Hospital of Nanjing Medical University, Suzhou, Jiangsu, China

**Keywords:** glucose metabolism, lipid metabolism, amino acid metabolism, abdominal aortic aneurysm, vascular smooth muscle cells

## Abstract

Abdominal aortic aneurysm (AAA) is often asymptomatic in its early stages, and rupture poses a life threatening risk. Currently, no effective pharmacological therapies are available, underscoring the importance of mechanistic research. Metabolic reprogramming—an adaptive process encompassing glucose, lipid, and amino acid metabolism—has increasingly gained attention in the context of AAA. These metabolic shifts, which coordinate cellular energy supply, biosynthesis, and signaling, critically shape vascular smooth muscle cell (VSMC) behavior, macrophage polarization, extracellular matrix remodeling, oxidative stress responses, and immune activation. Importantly, growing evidence suggests that crosstalk among these metabolic pathways orchestrates complex pathophysiological networks driving AAA initiation and progression. Exploring AAA pathogenesis from an integrated metabolic perspective not only helps elucidate underlying mechanisms but also provides new insights and potential therapeutic targets.

## 1 Introduction

Aneurysms, particularly abdominal aortic aneurysms (AAA), are common cardiovascular disorders characterized by localized dilation of the aortic wall. When persistent, they may progress to rupture, posing a life-threatening risk (Wanhainen et al., 2019). AAAs are typically asymptomatic, yet rupture is frequently fatal if surgical repair cannot be achieved in time. It has been reported that, in 2017, AAA accounted for approximately 167,200 deaths worldwide, with an estimated 3 million disability-adjusted life years (DALYs) lost (Wei et al., 2021). Each year, rupture of AAA leads to about 8,000 deaths in the United Kingdom and approximately 15,000 deaths in the United States. The disease is more prevalent in men, with an estimated prevalence of 1.3%–8.9%, compared to 1.0%–2.2% in women. The overall mortality of ruptured AAA ranges from 65% to 85%, with nearly half of the deaths occurring before patients reach the operating room (Sakalihasan et al., 2005). Most AAAs are nonspecific, with no clearly defined cause (Johnston et al., 1991). A minority of aneurysms have established etiologies, secondary to conditions such as atherosclerotic disease, trauma, connective tissue disorders (e.g., Marfan syndrome, Ehlers–Danlos syndrome type IV), infectious diseases (e.g., tuberculosis, syphilis, bacterial, or fungal infections), and inflammatory diseases (Sakalihasan et al., 2005). Although the mechanisms underlying aneurysm formation have been extensively investigated over the past decades, their exact pathophysiological processes remain incompletely understood.

Accumulating evidence suggests that metabolic reprogramming contributes to the onset and progression of cardiovascular diseases (Chiong et al., 2014; Hall et al., 2001). Metabolic reprogramming refers to the process by which cells alter their metabolic patterns to meet bioenergetic and biosynthetic demands, thereby promoting survival, proliferation, and growth. This encompasses glucose, lipid, and amino acid metabolism. Intracellular metabolic regulation of vascular smooth muscle cells (VSMCs) has been implicated in the pathogenesis of atherosclerosis, systemic hypertension, diabetes, pulmonary hypertension, vascular calcification, and aneurysm formation (Shi et al., 2020). In response to distinct stimuli, macrophages undergo a spectrum of transcriptional and proteomic changes that correspond to different phenotypic states. Classically activated macrophages (M1) exhibit a pro-inflammatory phenotype that accelerates AAA development by inducing inflammatory responses, secreting matrix metalloproteinases (MMPs) that promote extracellular matrix (ECM) degradation, upregulating peroxidase expression, and enhancing oxidative stress (Raffort et al., 2017). Collectively, emerging studies indicate that these metabolic alterations, acting through multicellular and multipathway mechanisms, critically modulate AAA progression. This provides multiple potential therapeutic targets and novel strategies for clinical intervention. Accordingly, this review focuses on recent advances regarding the pathological roles of metabolic reprogramming in the initiation and progression of AAA.

## 2 Pathophysiological basis of abdominal aortic aneurysm

The development of abdominal aortic aneurysm (AAA) is closely associated with alterations in the connective tissue of the aortic wall, in which elastic fibers and fibrillar collagens are the principal determinants of aortic mechanical properties (Melrose et al., 1998). Elastin and its associated proteins form an elastic fiber network that imparts viscoelasticity to the arterial wall; while intermolecular cross-links maintain structural stability, these fibers are simultaneously susceptible to degradation by elastolytic proteases. Elastic fibers are predominantly localized within the medial layer of the aorta, closely integrated with vascular smooth muscle cells (VSMCs), whereas collagens are abundantly distributed in both the medial and adventitial layers. Type I and type III collagens constitute the major components, conferring tensile strength and preserving vascular structural integrity. A hallmark pathological feature of AAA tissue is the fragmentation of elastic fibers and the depletion of elastin, a process that typically arises during the early stages of aneurysm expansion and continues up to rupture (Baxter et al., 1992; Sakalihasan et al., 1993). Loss of elastin represents an early event in aneurysm formation, while collagen degradation is a critical factor contributing to rupture. Matrix metalloproteinases (MMPs) play a central role in AAA progression; their excessive activation, combined with an imbalance against antiproteases, markedly accelerates vascular wall degradation, thereby promoting aneurysmal enlargement and rupture (Rao et al., 1996; Eriksson et al., 2004). In parallel, VSMCs exert dual functions during vascular remodeling: they not only synthesize various extracellular matrix proteins but also secrete proteases, thereby participating in the dynamic regulation of aortic structure (Lopez-Candales et al., 1997). Moreover, aneurysm progression is closely linked to intraluminal thrombus (ILT) formation. The processes of thrombus development and resolution can induce local hypoxia and trigger inflammatory responses, further driving aneurysm progression (Wang et al., 2014). Collectively, degradation of elastin and collagen, alterations in VSMC function, and the formation and remodeling of intraluminal thrombus represent the core mechanisms underlying the initiation and progression of abdominal aortic aneurysm.

## 3 Overview of metabolic reprogramming

Metabolic reprogramming refers to the process by which cells adapt to varying physiological or pathological conditions by altering their metabolic pathways to meet new demands. For example, in tumor cells, glucose metabolism is often shifted toward aerobic glycolysis—known as the Warburg effect—to support rapid proliferation (Wang et al., 2014). Similarly, immune cells, endothelial cells, and vascular smooth muscle cells undergo comparable metabolic reprogramming in response to different environmental stresses. Metabolic reprogramming not only influences cellular energy supply but also regulates cell function. Alterations in fatty acid metabolism, amino acid metabolism, and redox balance are closely associated with biological processes such as proliferation, migration, inflammatory responses, and apoptosis (Chen et al., 2024). Increasing evidence indicates that metabolic reprogramming plays a critical role in cardiovascular diseases, including atherosclerosis and heart failure.

## 4 Role of metabolic reprogramming in abdominal aortic aneurysm

### 4.1 Glucose metabolism

Genomic analyses have revealed that a prominent feature in patients with abdominal aortic aneurysm (AAA) as well as in the angiotensin II (Ang II) experimental model is metabolic reprogramming, characterized by enhanced glycolysis and suppressed glucose oxidative phosphorylation. Imaging studies have further demonstrated increased GLUT-mediated ^18F-fluorodeoxyglucose (^18F-FDG) uptake in AAA tissues, indicating elevated glucose metabolic activity within the lesions. Subsequent investigations showed that the glycolytic inhibitor 2-deoxy-D-glucose (2-DG) attenuates CaCl_2_-induced aortic dilation and reduces aneurysm formation in the Ang II model. This metabolic shift promotes the initiation and progression of AAA primarily by modulating the physiological and pathological functions of vascular smooth muscle cells and macrophages (Tsuruda et al., 2012).

#### 4.1.1 Vascular smooth muscle cells

In the pathogenesis and progression of abdominal aortic aneurysm (AAA), glucose metabolic reprogramming is recognized as one of the key molecular features. Vascular smooth muscle cells (VSMCs) represent the principal effector cells of this process, with glucose uptake primarily dependent on GLUT1 (Hruz and Mueckler, 2001) and regulated by signaling pathways such as Akt/mTOR (Lin et al., 2013). By promoting glycolysis and the tricarboxylic acid (TCA) cycle, this reprogramming accelerates glucose flux, thereby enhancing cellular proliferation and resistance to apoptosis. A phenomenon analogous to the Warburg effect in cancer cells has also been observed in VSMCs, wherein aerobic glycolysis is favored even under normoxic conditions (Warburg, 1956; Chen et al., 2018). Although energetically less efficient, this metabolic mode provides rapid ATP generation and abundant biosynthetic intermediates, thereby supporting cell proliferation, migration, and phenotypic switching (Yang et al., 2010; Pfeiffer et al., 2001). Within this context, key glycolytic enzymes (Jia et al., 2022; Jain et al., 2021) and lactate metabolism (Perez et al., 2010; Zhao et al., 2020) play central roles. Enhanced glycolysis mediated by pyruvate kinase isoform M2 (PKM2), together with lactate accumulation and altered transport, reshapes the intra- and extracellular metabolic milieu and further modulates VSMC phenotypic transformation and matrix remodeling via signaling pathways (Zhou et al., 2019; Zhang et al., 2022). Simultaneously, alterations in the pyruvate dehydrogenase kinase (PDK)/pyruvate dehydrogenase (PDH) balance redistribute glucose utilization between oxidative phosphorylation and glycolysis (Roche et al., 2001). In particular, upregulation of PDK4 has been linked not only to metabolic dysregulation (Zhu et al., 2018) but also to vascular calcification and impaired autophagy. Moreover, TCA cycle intermediates such as α-ketoglutarate (α-KG) have been proposed to exert protective effects through their antioxidant (Tian et al., 2020) and anti-inflammatory (Asadi Shahmirzadi et al., 2020) properties, mitigating AAA progression by reducing reactive oxygen species generation (Liu et al., 2022). In addition, diversion of glucose into the pentose phosphate pathway (PPP) supplies NADPH and nucleotides that support anabolic biosynthesis and antioxidative defense, thereby contributing to the survival and functional stability of VSMCs (Alamri et al., 2018; Ruiz-Ramírez et al., 2014; Dong et al., 2015). Overall, from glucose uptake to glycolysis, lactate metabolism, PDK/PDH regulation, PPP flux, and TCA cycle intermediates, glucose metabolic reprogramming shapes the functional state of VSMCs through multilayered mechanisms. These metabolic alterations not only drive the pathology of AAA but also highlight new directions for metabolic interventions and potential therapeutic targets.

#### 4.1.2 Macrophages

In the pathophysiology of abdominal aortic aneurysm (AAA), macrophage infiltration and polarization are considered pivotal events (Song et al., 2022; Dale et al., 2016; Batra et al., 2018). Classically activated M1 macrophages predominantly rely on glycolytic metabolism (Odegaard and Chawla, 2011). By enhancing glucose uptake, increasing lactate production, and elevating reactive oxygen species (ROS) generation (Fukuzumi et al., 1996), they drive inflammatory responses (Freemerman et al., 2014), extracellular matrix degradation, and oxidative stress, thereby accelerating structural injury of the aortic wall. In contrast, alternatively activated M2 macrophages preferentially utilize oxidative metabolism and exhibit anti-inflammatory and tissue-reparative properties. Their upregulation is regarded as a compensatory mechanism that helps restrain AAA expansion and rupture (Rateri et al., 2011). Metabolic reprogramming plays a central role in macrophage polarization. Upregulation of GLUT1 and regulation of the glycolytic enzyme pyruvate kinase isoform M2 (PKM2) (Gao et al., 2012) not only fuel proinflammatory cytokine production but also sustain the M1 phenotype through signaling pathways such as HIF-1 and STAT3. Conversely, downregulation of lactate dehydrogenase A (LDHA) can reduce lactate levels and attenuate inflammatory responses (Song et al., 2019). Tricarboxylic acid (TCA) cycle intermediates also exert bidirectional regulatory effects: for instance, succinate promotes proinflammatory cytokine expression, whereas α-ketoglutarate (α-KG) supports M2 polarization and anti-inflammatory gene expression through epigenetic remodeling (Liu et al., 2017). Although current pharmacological studies targeting glucose metabolism are primarily focused on oncology (Yang et al., 2021; Pelicano et al., 2006; Anderson et al., 2018), accumulating preclinical evidence suggests that targeting glycolysis, the TCA cycle, and oxidative phosphorylation may represent promising strategies for AAA intervention. Advancing this area of research will not only help elucidate the crosstalk between metabolism and immunity but also provide novel therapeutic avenues and potential targets for the prevention and treatment of AAA.

Recent studies further emphasize that macrophage polarization is not solely determined by single metabolic cues but by the integrated influence of glucose, fatty acid, and amino acid metabolism. For instance, succinate and α-ketoglutarate jointly regulate pro- and anti-inflammatory transcriptional programs, while fatty acid oxidation modulates HIF-1α activity and ROS production, creating a metabolic–immune feedback loop that sustains inflammatory microenvironments.

### 4.2 Lipid metabolism

Recent studies have demonstrated that long-chain polyunsaturated fatty acids (LCPUFAs), particularly ω-3 fatty acids such as eicosapentaenoic acid (EPA) and docosahexaenoic acid (DHA), are closely associated with the development and progression of abdominal aortic aneurysm (AAA) (Meital et al., 2019). Since humans cannot synthesize polyunsaturated fatty acids, their levels primarily depend on dietary intake. Clinical and randomized controlled trials have reported reduced EPA levels in patients with AAA, with both absolute EPA concentration and the EPA/arachidonic acid (ARA) ratio showing significant inverse correlations with aneurysm diameter and growth rate (Aikawa et al., 2017). Mechanistically, dietary supplementation with EPA and DHA enriches cell membrane phospholipids with ω-3 fatty acids, reduces the generation of ARA and its pro-inflammatory metabolites (e.g., PGE2, TXA2, LTB4), and suppresses macrophage-mediated inflammatory responses, thereby exerting protective effects against AAA progression (Yoshihara et al., 2015). Conversely, ARA, as an ω-6 fatty acid, aggravates disease through its pro-inflammatory actions (Ricciotti and FitzGerald, 2011; Soto et al., 2018). Animal studies further show that inhibition of the COX pathway improves the structural integrity of vascular elastin and downregulates matrix metalloproteinase (MMP) expression (Guo et al., 2013). Observational studies suggest that low-dose aspirin may slow the growth of medium-sized AAAs; however, no randomized controlled trials have yet confirmed the efficacy of any pharmacological agent in stabilizing or halting AAA expansion (Lindholt et al., 2008). Overall, the anti-inflammatory and immunomodulatory properties of LCPUFAs provide novel insights into the prevention and treatment of AAA, though further high-quality evidence is required to support clinical application. At present, the role of fatty acid metabolism in AAA pathogenesis remains incompletely understood. Metabolomic analyses indicate aberrant lipid metabolism in AAA tissues (Xie et al., 2023; Zhang et al., 2023), and elevated serum platelet-derived growth factor (PDGF) levels in patients can promote fatty acid oxidation in vascular smooth muscle cells (VSMCs) by upregulating carnitine palmitoyl transferase 1 (CPT1). This enhances cell proliferation and inhibits apoptosis-related pathways, suggesting that fatty acid oxidation may contribute to vascular remodeling (Yuwen et al., 2019). In macrophages, however, the role of fatty acid oxidation is controversial: some studies suggest that it suppresses inflammation and lipid accumulation (Malandrino et al., 2015; Namgaladze et al., 2014), while others indicate that CPT deficiency may confer anti-inflammatory and anti-atherogenic effects (Nomura et al., 2019). In addition, fatty acid biosynthesis has attracted attention in the context of VSMC phenotypic switching. In synthetic VSMCs, fatty acid synthase is upregulated, and altered activity of stearoyl-CoA desaturase 1 (SCD1) affects lipid composition and vascular metabolic homeostasis.

### 4.3 Amino acid metabolism

#### 4.3.1 Sulfur-containing amino acids

Cysteine (Cys), methionine (Met), and their metabolic derivative homocysteine (Hcy) play important roles in the context of abdominal aortic aneurysm (AAA) (Zaric et al., 2019). Hyperhomocysteinemia accelerates vascular wall degradation and aneurysmal expansion by increasing reactive oxygen species (ROS) generation, depleting nitric oxide (NO), upregulating matrix metalloproteinase (MMP) activity, and promoting the phenotypic transition of vascular smooth muscle cells (VSMCs) from a contractile to a synthetic state (Steed and Tyagi, 2011). Clinically, folate, vitamins B6/B12, and methionine-restricted diets have been shown to reduce Hcy levels, suggesting their potential for therapeutic intervention (Warsi et al., 2004). In addition, novel agents such as cystathionine β-synthase (CBS) modifiers (e.g., OT-58) hold promise as future targeted strategies (Bublil and Majtan, 2020).

#### 4.3.2 Tryptophan

Tryptophan (Trp) metabolism through the kynurenine pathway (KP) is upregulated in abdominal aortic aneurysm (AAA) tissues, with increased expression of key enzymes and metabolites (Nishimura et al., 2021). Indoleamine 2,3-dioxygenase (IDO)-mediated Trp metabolism promotes inflammation, matrix metalloproteinase (MMP) expression (Wang et al., 2017), and apoptosis, whereas its metabolite 5-methoxytryptophan (5-MTP) exhibits vasoprotective and anti-inflammatory effects (Wu et al., 2020; Yang et al., 2015). Animal studies have demonstrated that inhibition of the KP can delay AAA formation, and IDO inhibitors have already been applied in other diseases (Watanabe et al., 2018); however, clinical validation in vascular disorders remains lacking (Song et al., 2017; Ramprasath et al., 2021). Notably, KP metabolism is closely associated with the aging process, which aligns with the strong age dependence of AAA, underscoring its potential significance for future research.

#### 4.3.3 Taurine

Taurine (Tau) exerts significant vasoprotective effects through its antioxidant activity, scavenging of oxidants such as hypochlorous acid (HClO), reduction of inflammatory cell infiltration, and inhibition of matrix metalloproteinase (MMP) activity (Bkaily et al., 2020; Kim et al., 2017; Chao de la Barca et al., 2022). Animal studies have demonstrated that taurine supplementation effectively suppresses angiotensin II (Ang II)-induced AAA formation (Brethel et al., 2023). Moreover, its regulatory roles in VSMC migration, anti-apoptotic responses, and anti-calcification suggest its involvement in maintaining vascular wall homeostasis (Korshunov et al., 2006). However, clinical data in patients with AAA are currently lacking, and its protective effects remain to be further validated.

#### 4.3.4 Glycine

Glycine (Gly) plays an important role in antioxidant and anti-inflammatory processes by restoring glutathione synthesis, inhibiting reactive oxygen species (ROS) production, and suppressing NF-κB activation, thereby alleviating vascular inflammation (Ruiz-Ramírez et al., 2014; Chao de la Barca et al., 2022). Metabolomic analyses have revealed decreased serum glycine levels in AAA models, which may diminish its protective effects. In addition, glycine has been shown to reduce blood lipid levels (Rom et al., 2017), thereby mitigating the adverse impact of hyperlipidemia on AAA. However, its protective role has not yet been clinically validated.

#### 4.3.5 Glutamine and glutamate

Glutamine (Gln) serves as a critical substrate for cellular proliferation. It is transported into cells via the high-affinity L-Gln transporter solute carrier family 1 member 5 (SLC1A5), where it activates the mTORC1 signaling pathway and promotes the proliferation of vascular smooth muscle cells (VSMCs) (Osman et al., 2019). Both glutamate and glutamine also contribute to nitric oxide (NO) synthesis, with NO exerting protective effects by maintaining extracellular matrix homeostasis and promoting vasodilation (Zhang et al., 2003; Zhou et al., 2023). However, under conditions of metabolic dysregulation, excessive NO may induce vascular injury, suggesting a bidirectional role. Clinical studies have shown that glutamine supplementation can improve nitrogen balance and immune status following aortic surgery; nevertheless, its potential role in the prevention or treatment of AAA requires further investigation (Brinkmann et al., 2016).

#### 4.3.6 Branched-chain amino acids

**FIGURE 1:**
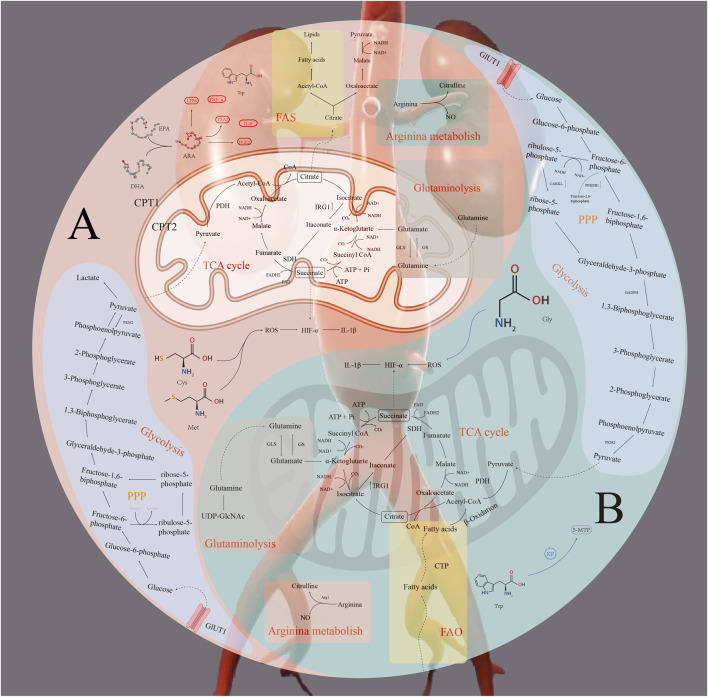
Overview of the mechanism of abdominal aortic aneurysm.

Branched-chain amino acid (BCAA) levels, as well as their ratios with Gly and Gln, have been identified through metabolomic analyses as potential biomarkers of AAA (Zaric et al., 2020). Leucine supplementation has been shown to improve macrophage lipid metabolism, enhance mitochondrial function, attenuate inflammation, and improve vascular elasticity. Clinical studies indicate that leucine-rich diets enhance cardiometabolic health in older adults; however, direct evidence supporting a protective role in AAA remains lacking (Kirk et al., 2021). All of the above-described mechanisms of metabolic reprogramming are summarized in Figure 1. Table 1 provides an overview summarizing the main metabolic pathways involved in abdominal aortic aneurysm (AAA) as well as the key molecules and targets.

**TABLE 1:** Key molecules and targets in the pathway of abdominal aortic aneurysm.

Pathway	Key Molecules	Cellular Effects	Experimental/Clinical Evidence	Therapeutic Potential
Glycolysis	GLUT1, PKM2, PDK4	Proliferation, ROS production, M1 polarization	Preclinical (mouse, human tissue)	PKM2 inhibitors, 2-DG
Fatty acid oxidation	CPT1, SCD1	VSMC remodeling, inflammatory regulation	Preclinical	CPT1 inhibitors, FAO modulators
Amino acid metabolism	IDO, α-KG, 5-MTP	Epigenetic regulation, apoptosis, M2 polarization	Preclinical/limited clinical	IDO inhibitors, α-KG supplementation

Figure 1 provides an overview of metabolic reprogramming in abdominal aortic aneurysm (AAA), illustrating glucose, lipid, and amino acid metabolism on the basis of M1 and M2 macrophage phenotypes. A: M1 macrophage; B: M2 macrophage; FAS: fatty acid synthesis; FAO: fatty acid oxidation.

### 4.4 Integrative perspective on metabolic reprogramming

Metabolic reprogramming represents a dynamic and interconnected network that orchestrates cellular adaptation to pathological stimuli. Rather than functioning as isolated processes, glucose, lipid, and amino acid metabolism are tightly integrated: for example, glycolytic intermediates fuel fatty acid synthesis, lipid oxidation regulates inflammatory tone and mitochondrial function, and amino acid–derived metabolites modulate epigenetic states that influence macrophage polarization. This interdependence suggests that AAA progression results from the synergistic actions of metabolic pathways, highlighting the importance of a systems-level view when considering therapeutic interventions.

## 5 Therapy

Given the complex interplay between metabolism and inflammation, targeting immune metabolism holds significant therapeutic potential, though relevant strategies remain in their early stages. The metabolic reprogramming of artery-resident macrophages provides a promising avenue for further investigation, opening new strategies for cardiovascular disease treatment. We propose that inhibiting glycolysis and the fatty acid synthesis (FAS) pathway in M1 macrophages, or enhancing fatty acid oxidation (FAO) in M2 macrophages, may effectively reduce foam cell formation, mitigate inflammation, and slow the progression of atherosclerosis (Liu et al., 2021; Hou et al., 2023). Future studies should further elucidate the mechanisms of macrophage metabolic reprogramming in atherosclerosis, as they may yield highly specific therapeutic targets capable of improving plaque stability, reducing inflammation, and significantly enhancing clinical outcomes. This approach also provides a solid theoretical and practical foundation for the treatment of abdominal aortic aneurysm (An et al., 2025). In addition, dimethyl fumarate (DMF), a derivative of the tricarboxylic acid cycle, suppresses aerobic glycolysis in immune cells by modifying cysteine residues (e.g., GAPDH), thereby inducing macrophage polarization toward an anti-inflammatory phenotype. This results in improved ventricular remodeling, reduced collagen deposition, and enhanced angiogenesis following myocardial infarction, while also alleviating myocardial injury in diabetic models, demonstrating cardiovascular protective effects (Mouton et al., 2021; Bresciani et al., 2023). Similarly, the immunometabolite itaconate, generated from cis-aconitate via CAD (encoded by IRG), competitively inhibits succinate dehydrogenase (SDH), decreases mitochondrial ROS and pro-inflammatory gene expression (downregulating IL-1β and IL-6; upregulating IL-1RA and IL-10), and modifies glycolytic enzymes such as GAPDH, ALDOA, and LDHA to inhibit glycolysis, thereby exerting protective effects in murine models of myocardial infarction and doxorubicin-induced cardiotoxicity. Moreover, rapamycin inhibits mTORC1, thereby reducing glycolysis and inflammatory polarization, limiting post-MI macrophage infiltration, and improving outcomes (Shan et al., 2024; Shan et al., 2019; Sciarretta et al., 2014). Collectively, these findings indicate that multiple metabolism-targeting agents hold promise for the treatment and prevention of cardiovascular diseases, yet further studies are needed to elucidate underlying mechanisms, identify precise targets, and advance innovative therapeutic strategies into clinical practice.

## 6 Clinical translation and therapeutic perspectives

While numerous metabolic targets (e.g., PKM2, PDK4, IDO, CPT1) and modulators (e.g., DMF, itaconate, rapamycin) show promise in preclinical models, significant barriers remain in translating these findings into clinical therapies. Challenges include metabolic heterogeneity across patient populations, off-target effects of systemic metabolic modulators, and a lack of reliable biomarkers to monitor metabolic changes *in vivo*. To bridge this gap, future studies should: (1) develop cell-specific metabolic interventions, (2) integrate metabolomics with single-cell and spatial omics to stratify patients by metabolic phenotype, and (3) conduct prospective clinical trials assessing safety, efficacy, and biomarker-guided treatment responses.

## 7 Future directions

Despite major advances, several critical questions remain unresolved. Future research should: Characterize metabolic heterogeneity across VSMC subpopulations and macrophage subsets using spatial transcriptomics and metabolomics. Investigate how aging reshapes metabolic networks, given the age dependence of AAA. Explore metabolic–immune crosstalk in the context of systemic comorbidities (e.g., diabetes, dyslipidemia). Design combinatorial therapies targeting multiple metabolic pathways simultaneously to maximize therapeutic efficacy.

## 8 Conclusion

Abdominal aortic aneurysm (AAA) is associated with extremely high mortality due to its insidious onset and the lack of effective interventions. Metabolic reprogramming plays a pivotal role in AAA initiation and progression, influencing vascular wall homeostasis through intertwined metabolic networks that regulate inflammation, oxidative stress, apoptosis, and phenotypic switching. Integrating these insights into clinical strategies requires deeper mechanistic understanding, robust biomarker development, and innovative therapeutic design. Ultimately, viewing AAA through the lens of metabolic systems biology may unlock transformative avenues for individualized prevention and treatment.
